# Construction of a pyroptosis-related lncRNAs signature for predicting prognosis and immunotherapy response in glioma

**DOI:** 10.1097/MD.0000000000032793

**Published:** 2023-02-10

**Authors:** Qianrong Huang, Jun Yan, Qian Jiang, Fangzhou Guo, Ligen Mo, Teng Deng

**Affiliations:** a Department of Neurosurgery, Guangxi Medical University Cancer Hospital, Nanning, Guangxi, PR China.

**Keywords:** glioma, immunotherapy, lncRNA, prognosis, pyroptosis, signature

## Abstract

Recent studies have proved that pyroptosis-related long non-coding RNAs (PRlncRNAs) are closely linked to tumor progression, prognosis, and immunity. Here, we systematically evaluated the correlation of PRlncRNAs with glioma prognosis. This study included 3 glioma cohorts (The Cancer Genome Atlas, Chinese Glioma Genome Atlas, and Gravendeel). Through Pearson correlation analysis, PRlncRNAs were screened from these 3 cohorts. Univariate Cox regression analysis was then carried out to determine the prognostic PRlncRNAs. A pyroptosis-related lncRNAs signature (PRLS) was then built by least absolute shrinkage and selection operator and multivariate Cox analyses. We systematically evaluated the correlation of the PRLS with the prognosis, immune features, and tumor mutation burden in glioma. A total of 14 prognostic PRlncRNAs overlapped in all cohorts and were selected as candidate lncRNAs. Based on The Cancer Genome Atlas cohort, a PRLS containing 7 PRlncRNAs was built. In all cohorts, the PRLS was proved to be a good predictor of glioma prognosis, with a higher risk score related to a poorer prognosis. We observed obvious differences in the immune microenvironment, immune cell infiltration level, and immune checkpoint expression in low- and high-risk subgroups. Compared with low-risk cases, high-risk cases had lower Tumor Immune Dysfunction and Exclusion scores and greater tumor mutation burden, indicating that high-risk cases can be more sensitive to immunotherapy. A nomogram combining PRLS and clinical parameters was constructed, which showed more robust and accurate predictive power. In conclusion, the PRLS is a potentially useful indicator for predicting prognosis and response to immunotherapy in glioma. Our findings may provide a useful insight into clinically individualized treatment strategies for patients.

## 1. Introduction

Glioma, the most prevalent brain malignancy, occurs at all ages and still has a high rate of recurrence and mortality due to aggressive growth and treatment resistance.^[1,2]^ The World Health Organization classifies gliomas into grades I to IV, and the higher the grade, the higher the malignancy.^[3]^ Grade IV gliomas, also known as glioblastoma (GBM), are known for their extreme aggressiveness and are the most common type with the shortest clinical prognosis, with a 5-year survival rate of <10%.^[1,4]^ Prognosis for lower-grade glioma (grade II–III) varies widely, with some patients exhibiting high sensitivity to treatment, but a significant proportion of patients can rapidly progress to GBM.^[5,6]^ Although surgery, radiotherapy, and chemotherapy are routinely used for glioma, patients’ clinical outcomes do not improve significantly.^[7,8]^ Therefore, further efforts in exploring prognostic models, potential therapeutic targets, and novel therapeutic strategies are of great clinical significance for this refractory tumor.

Pyroptosis is a kind of programmed cell death mediated by gasdermins, which is distinct from apoptosis and ferroptosis and is characterized by rapid breakdown of cell membranes and release of pro-inflammatory mediators.^[9,10]^ Recently, many studies have confirmed that pyroptosis is strongly associated with the occurrence and development of cancer.^[11–13]^ Treatment by inducing pyroptosis is expected to be a new method to break through the current dilemma of cancer therapy.^[14,15]^ Long non-coding RNAs (lncRNAs) comprise at least 200 nucleotides with no protein-coding function.^[16,17]^ The lncRNA not only mediate multiple biological functions, but also participate in the regulation of many key mechanisms in cancer.^[18–20]^ With the rapid development of bioinformatics, many kinds of specific lncRNAs have been identified in cancer, and the pyroptosis-related lncRNA (PRlncRNA) is one of them. Previous evidence has found that PRlncRNAs are linked to tumor progression, prognosis, and immune status.^[21–24]^ However, little is known about the function and mechanism of these small molecules in glioma.

In this paper, The Cancer Genome Atlas (TCGA), Chinese Glioma Genome Atlas (CGGA), and Gravendeel datasets were used to systematically explore the prognostic role of PRlncRNAs in glioma. We first identified overall survival (OS)-related PRlncRNAs in glioma, and creatively built and validated a prognostic pyroptosis-related lncRNAs signature (PRLS). Besides, we evaluated the association of PRLS with immune status and tumor mutation burden (TMB). Finally, we developed a PRLS-based nomogram to increase the accuracy of prognostic prediction for clinical patients. The purpose of the study was to elucidate the role of PRlncRNAs in glioma prognosis and provide useful insights into patient individualized therapy.

## 2. Materials and methods

This study was conducted using publicly available data and therefore did not require ethics committee approval.

### 2.1. Identification of PRlncRNAs

In this study, we obtained clinical and RNA-sequencing (fragment per kilobase million) data of TCGA and CGGA cohorts from TCGA and CGGA websites. We then converted the fragment per kilobase million data using log2 (*x* + 1) transformation. The clinical information and normalized microarray data of Gravendeel cohort were obtained from the GlioVis website. The inclusion criteria for glioma cases were: grade II to IV glioma; the OS time was ≥30 days; and the expressed data was available. A total of 33 pyroptosis-related genes were obtained from previous literatures.^[25,26]^ Next, based on the expression data, Pearson correlation analysis was performed to assess the relationship of 33 pyroptosis-related genes with lncRNAs. We set the criterion for PRlncRNAs as correlation coefficient |R| > 0.4, and *P* < .001.

### 2.2. PRLS establishment and validation

Next, we identified OS-related PRlncRNAs in all cohorts by univariate Cox analysis. Shared prognostic PRlncRNAs from these 3 cohorts were selected, which were considered to be the most accurate. Least absolute shrinkage and selection operator (LASSO) analysis was performed to narrow the range of shared PRlncRNAs in the training (TCGA) cohort. Multivariate Cox analysis was then utilized to build a prognostic PRLS. Risk scores for glioma cases were calculated by expression and regression coefficients of selected PRlncRNAs. The formula of the risk score was:

Risk score = (lncRNA1expression * lncRNA1Coef) + (lncRNA2expression * lncRNA2Coef) + … + (lncRNAnexpression * lncRNAnCoef).

After the hub PRlncRNAs were identified, we explored the correlation between their expression. In addition, a lncRNA-mRNA network of hub PRlncRNAs and related pyroptosis-related genes was established through Cytoscape software (version 3.8.0) (https://cytoscape.org/). We divided glioma cases into 2 subgroups according to the median risk score. We next carried out Kaplan–Meier (K-M) method and time-dependent receiver operating characteristic (tdROC) curve in all cohorts to evaluate the performance of the PRLS to predict patients’ OS. In addition, we investigated its prognostic value in patients receiving radiotherapy or chemotherapy in the CGGA cohort.

### 2.3. Enrichment analysis of the PRLS

By applying the “Rtsne” package in R, we conducted principal component analysis to evaluate the discriminating ability of the PRLS. Using the “limma” package, differentially expressed genes (DEGs) were identified in the 2 subgroups in training cohort, according to the following standards: |log_2_FC| > 1 and FDR < 0.05. Then we conducted Gene Ontology and Kyoto Encyclopedia of Genes and Genomes pathway analyses through the “clusterProfiler” package and set the range of significance as *P* < .05.

### 2.4. Evaluation of immune status

By applying the “estimate” package, we performed the ESTIMATE method to evaluate the immune score, stromal score, ESTIMATE score, and tumor purity of TCGA glioma cases.^[27]^ The CIBERSORT method was then utilized to assess the ratio of immune cell types, and the abundance of each immune cell type was compared between the 2 subgroups.^[28]^ Besides, the expression levels of immune checkpoints were compared in 2 subgroups. The Tumor Immune Dysfunction and Exclusion (TIDE) algorithm is a novel method to assess the therapeutic response of immune checkpoint inhibitors (ICIs), which has been well illustrated in recent researches.^[29,30]^ Here, we upload expression data of the TCGA glioma dataset to TIDE database to collect the corresponding score. We then utilized the TIDE algorithm to assess the response to ICIs in glioma cases with different PRLS scores.

### 2.5. TMB analysis

Glioma mutation data (TCGA-GBM and TCGA-lower-grade glioma) were obtained from the TCGA website. The evaluation and visualization of somatic variation data were carried out via the “maftools” package.^[31]^ The TMB for each glioma case was equal to the whole mutations divided by the whole length of exons. We compared TMB levels in the 2 risk subgroups and further assessed the relationship of TMB with PRLS scores using Spearman analysis. The optimal cutoff value for the 2 TMB subgroups was determined through the “survminer” package. Furthermore, we analyzed OS differences among different subgroups in combination with TMB and PRLS scores.

### 2.6. Establishment of a prognostic nomogram

Next, to assess if the PRLS had an independent prognostic effect, univariate/multivariate Cox analysis was carried out in combination with other clinical parameters of glioma. For further clinical application, we built a prognostic nomogram via the “rms” package according to independent prognostic parameters.

### 2.7. Statistical analysis

Chi-square test or Wilcox test was carried out for comparison between groups. Log-rank test was employed to assess the difference in prognosis between groups. Other statistical methods had been introduced above. Data analysis of our study was carried out by R software (v3.6.3) (https://www.r-project.org). *P* < .05 meant the difference was statistically significant.

## 3. Results

### 3.1. Screening of prognostic PRlncRNAs

**Figure 1.**
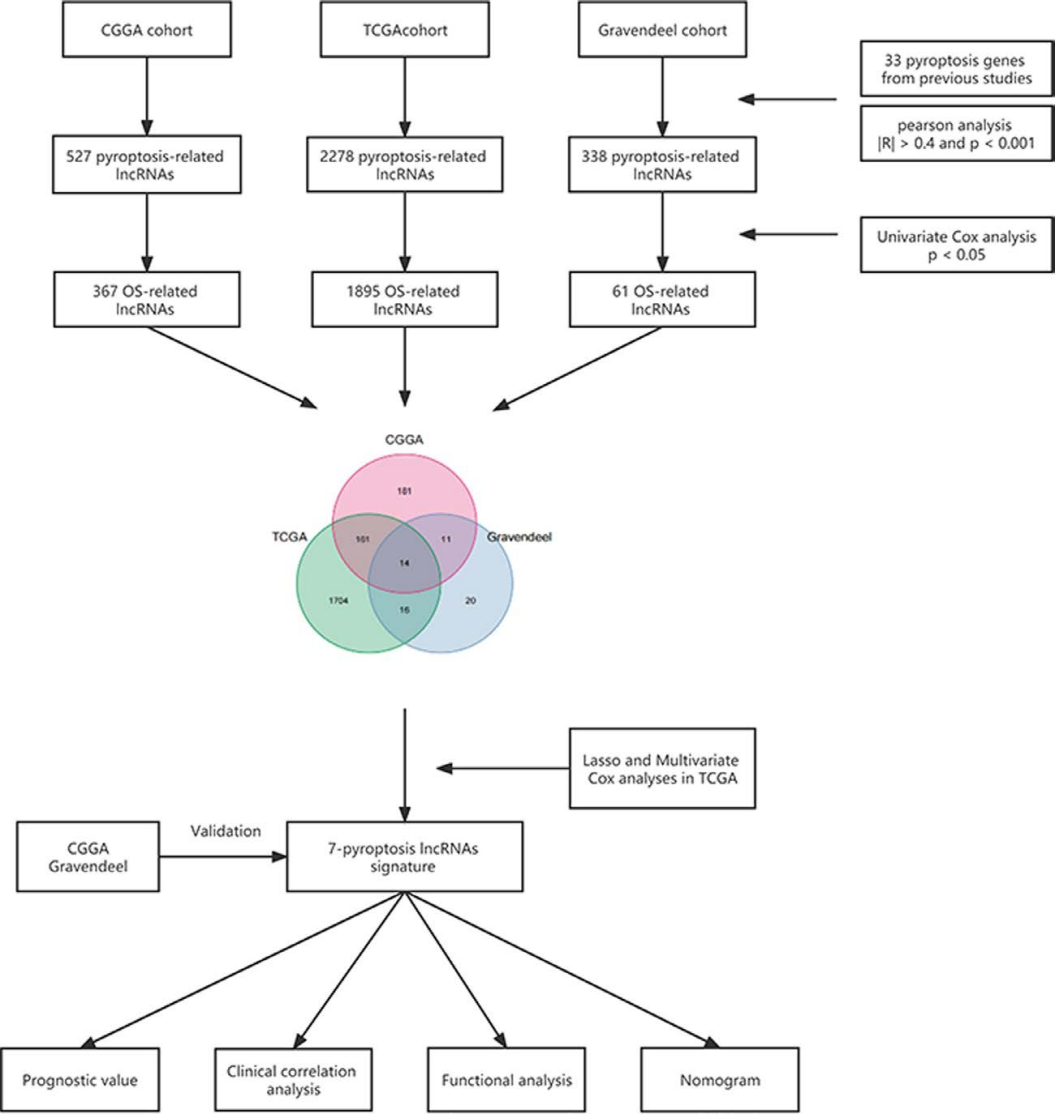
The flow chart of the study.

**Figure 2.**
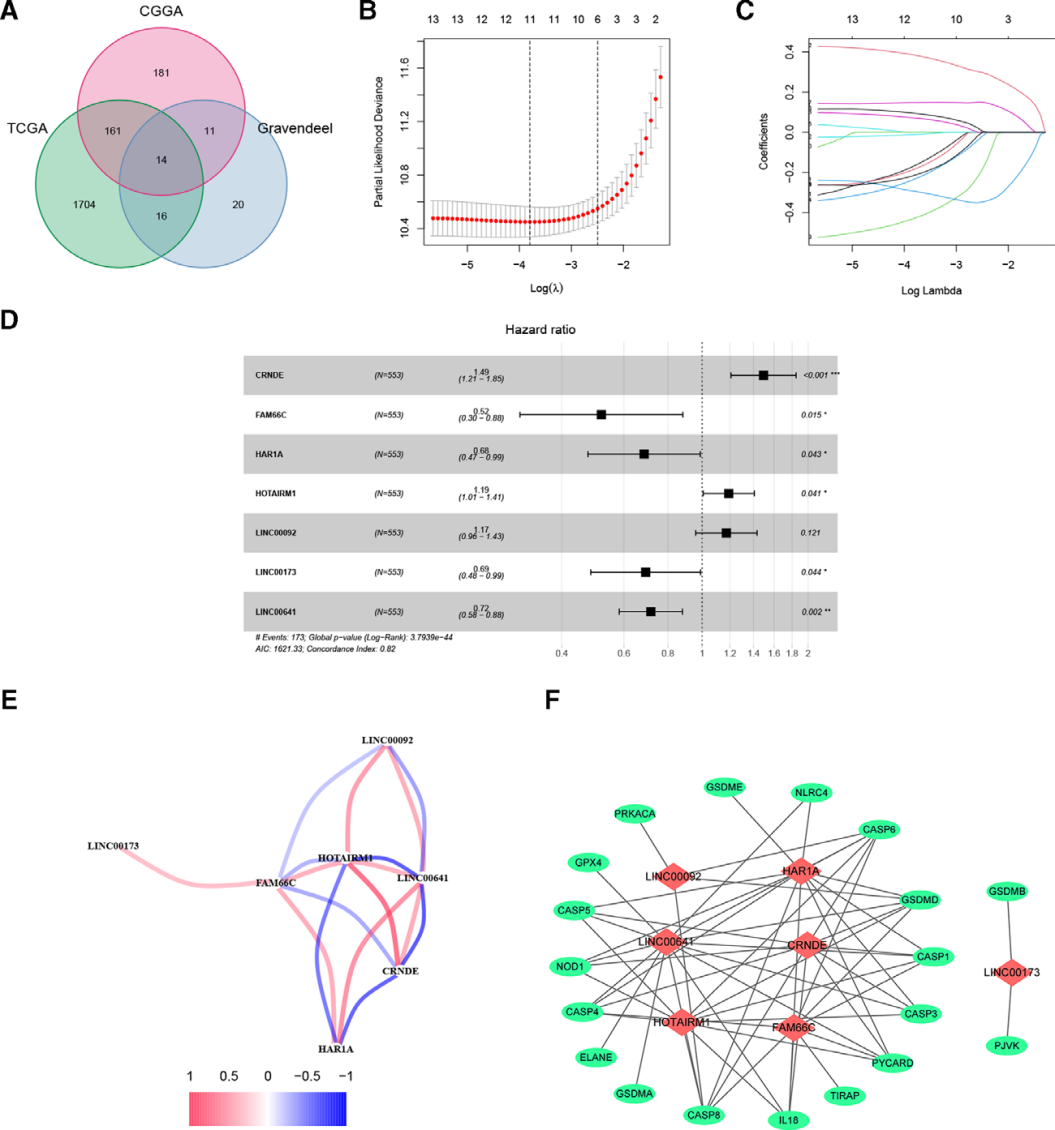
Identification of prognostic PRlncRNAs and PRLS building. (A) Shared prognostic PRlncRNAs of 3 cohorts were identified. (B–C) Based on TCGA cohort, LASSO regression method was utilized to reduce the risk of overfitting. (D) A PRLS was built by multivariate regression analysis. (E) The expression correlation among these 7 hub PRlncRNAs. (F) The co-expression network of these 7 lncRNAs and relevant pyroptosis-related genes. LASSO = least absolute shrinkage and selection operator, PRlncRNAs = pyroptosis-related long non-coding RNAs, PRLS = pyroptosis-related lncRNAs signature, TCGA = The Cancer Genome Atlas.

The whole flow chart of our study is shown in Figure 1. A total of 1765 glioma cases were eligible for inclusion (Table S1, Supplemental Digital Content, http://links.lww.com/MD/I406). Through Pearson analysis, we identified 2278 PRlncRNAs in TCGA, 527 in CGGA, and 338 in Gravendeel. We then obtained 1895, 367 and 61 OS-related PRlncRNAs in these 3 cohort by univariate Cox regression analysis, respectively. Finally, a total of 14 shared prognostic PRlncRNAs from 3 cohorts were identified and used as candidate lncRNAs (Fig. 2A).

### 3.2. PRLS building

To reduce the risk of overfitting, we performed LASSO analysis in training cohort (Fig. 2B and C). The number of PRlncRNAs was narrowed down to 11, which were then included in multivariate Cox regression analysis. Eventually, a PRLS containing 7 PRlncRNAs (CRNDE, FAM66C, HAR1A, HOTAIRM1, LINC00092, LINC00173, and LINC00641) was established (Fig. 2D and Table 1). The formula of the risk score was: risk score = (0.4019 * CRNDEexpression) + (-0.6580 * FAM66Cexpression) + (−0.3786 * HAR1Aexpression) + (0.1743 * HOTAIRM1expression) + (0.1588 * LINC00092expression) + (−0.3685 * LINC00173expression) + (−0.3346 * LINC00641expression). The expression correlation among these 7 hub PRlncRNAs is shown in Figure 2E. In addition, the mRNA-lncRNA co-expression network showed their relationship with the corresponding pyroptosis-related genes (Fig. 2F).

**Table 1:** The PRlncRNAs in the signature and the corresponding coefficients.

ID	Coef	HR	HR.95L	HR.95H	*P* value
CRNDE	0.4019	1.4947	1.2082	1.8492	<.001
FAM66C	−0.6580	0.5179	0.3041	0.8819	.015
HAR1A	−0.3786	0.6848	0.4744	0.9885	.043
HOTAIRM1	0.1743	1.1904	1.0074	1.4067	.041
LINC00092	0.1588	1.1722	0.9590	1.4326	.121
LINC00173	−0.3685	0.6918	0.4832	0.9903	.044
LINC00641	−0.3346	0.7156	0.5820	0.8799	.002

HR = hazard ratio, PRlncRNAs = pyroptosis-related long non-coding RNAs.

### 3.3. Predictive value of the PRLS

**Figure 3.**
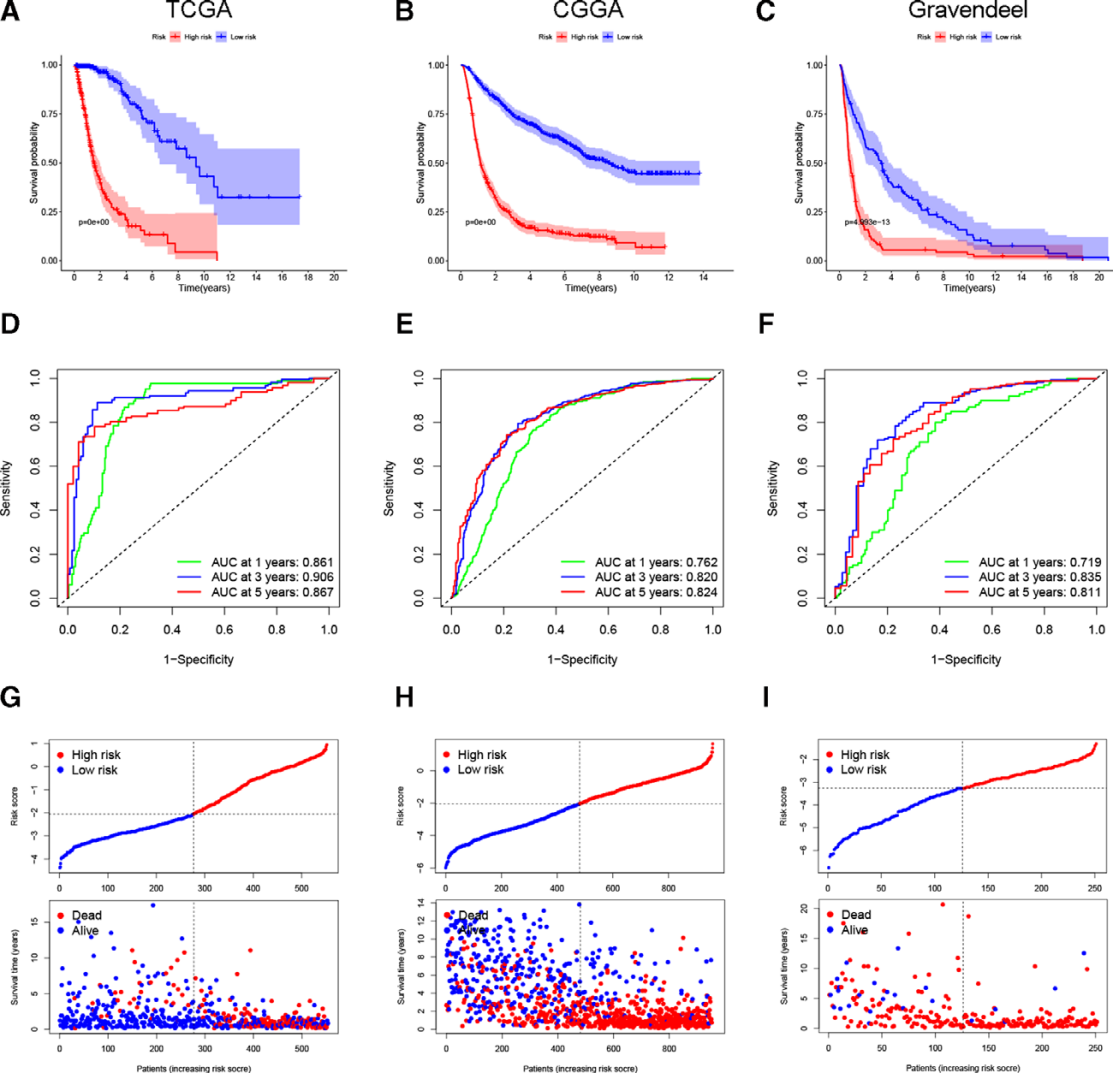
The prognostic role of the PRLS in glioma. (A–C) K-M survival analysis of the PRLS. (D–F) ROC curves of the PRLS for predicting glioma survival. (G–I) Relationship of risk score with survival time and survival status. K-M = Kaplan–Meier, PRLS = pyroptosis-related long non-coding RNAs signature, ROC = receiver operating characteristic.

According to the above formula, we obtained the risk score for each glioma case, with the median score as the cutoff point for the 2 risk subgroups. K-M survival analyses in all 3 cohorts showed that high-risk cases had a worse clinical outcome than low-risk cases (Fig. 3A–C). The tdROC curves showed that area under the curves for predicting survival rates at 1, 3, and 5 years were >0.7 in all cohorts (Fig. 3D–F). It was clear from the risk score, survival status, and OS distribution diagram that the OS was shortened and mortality was increased in high-risk patients (Fig. 3G–I).

**Figure 4.**
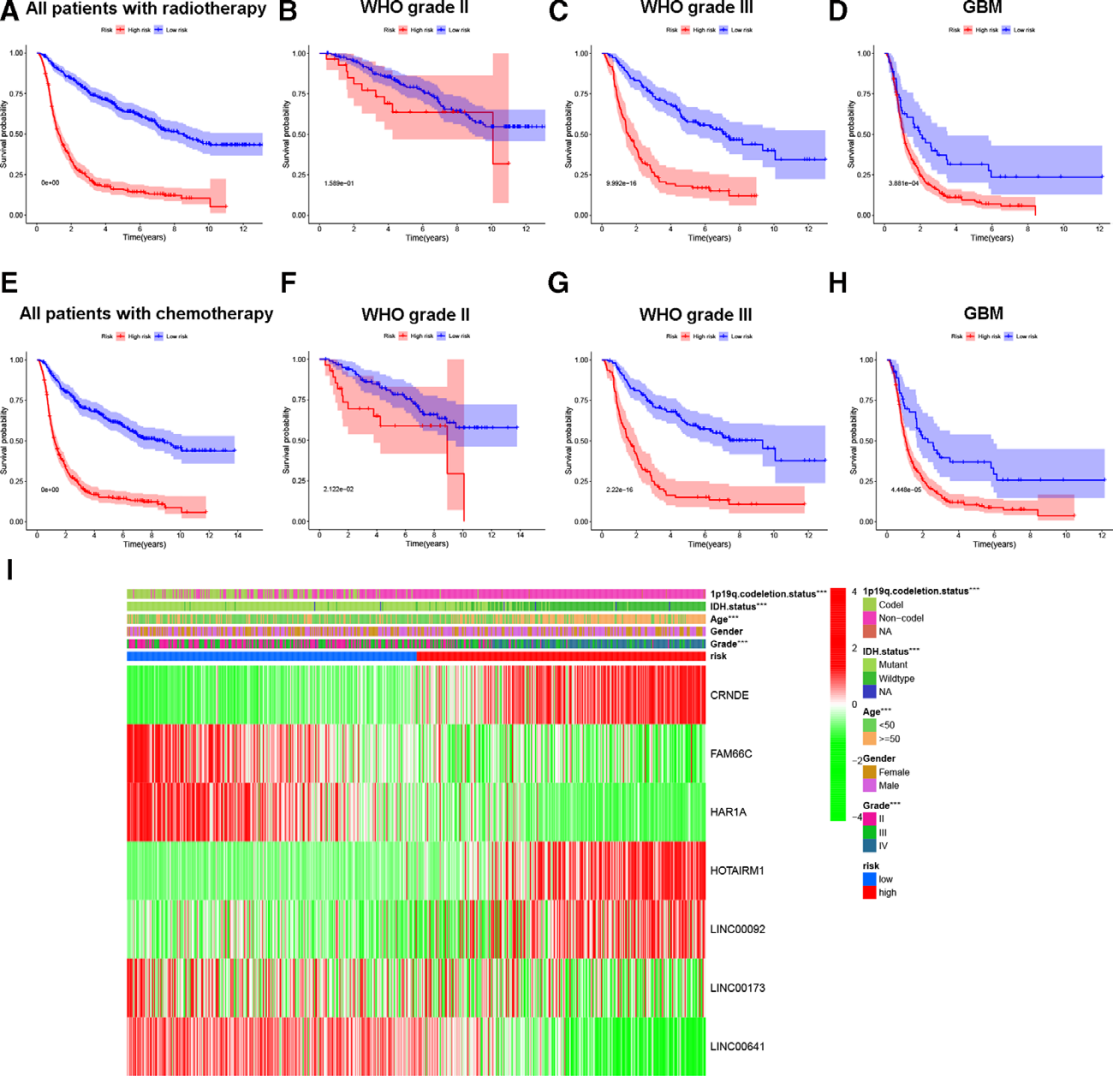
The prognostic role of the PRLS in patients who received (A–D) radiotherapy and (E–H) chemotherapy in the CGGA cohort. (I) Association of the PRLS with clinical features in the TCGA cohort. CGGA = Chinese Glioma Genome Atlas, PRLS = pyroptosis-related long non-coding RNAs signature, TCGA = The Cancer Genome Atlas.

In view of the strong predictive efficacy of the PRLS, we analyzed its predictive power in glioma cases receiving radiotherapy or chemotherapy. The results showed that for all patients who received radiotherapy, the prognosis of low-risk patients was better than that of high-risk patients (Fig. 4A). In subgroup analyses, we observed the same trend in both grade III patients and GBM patients (Fig. 4C and D), whereas there was no obvious difference for grade II patients (Fig. 4B). For chemotherapy, both whole cohort and subgroup analyses showed that compared with high-risk cases, low-risk cases had longer OS (Fig. 4E–H). In addition, we found significant differences in clinical features between the 2 risk subgroups. As shown in Figure 4I, patients with higher grades, age ≥50 years, IDH wild-type, and 1p19q non-codeletion were mainly concentrated in the high-risk group.

### 3.4. Functional enrichment analysis

**Figure 5.**
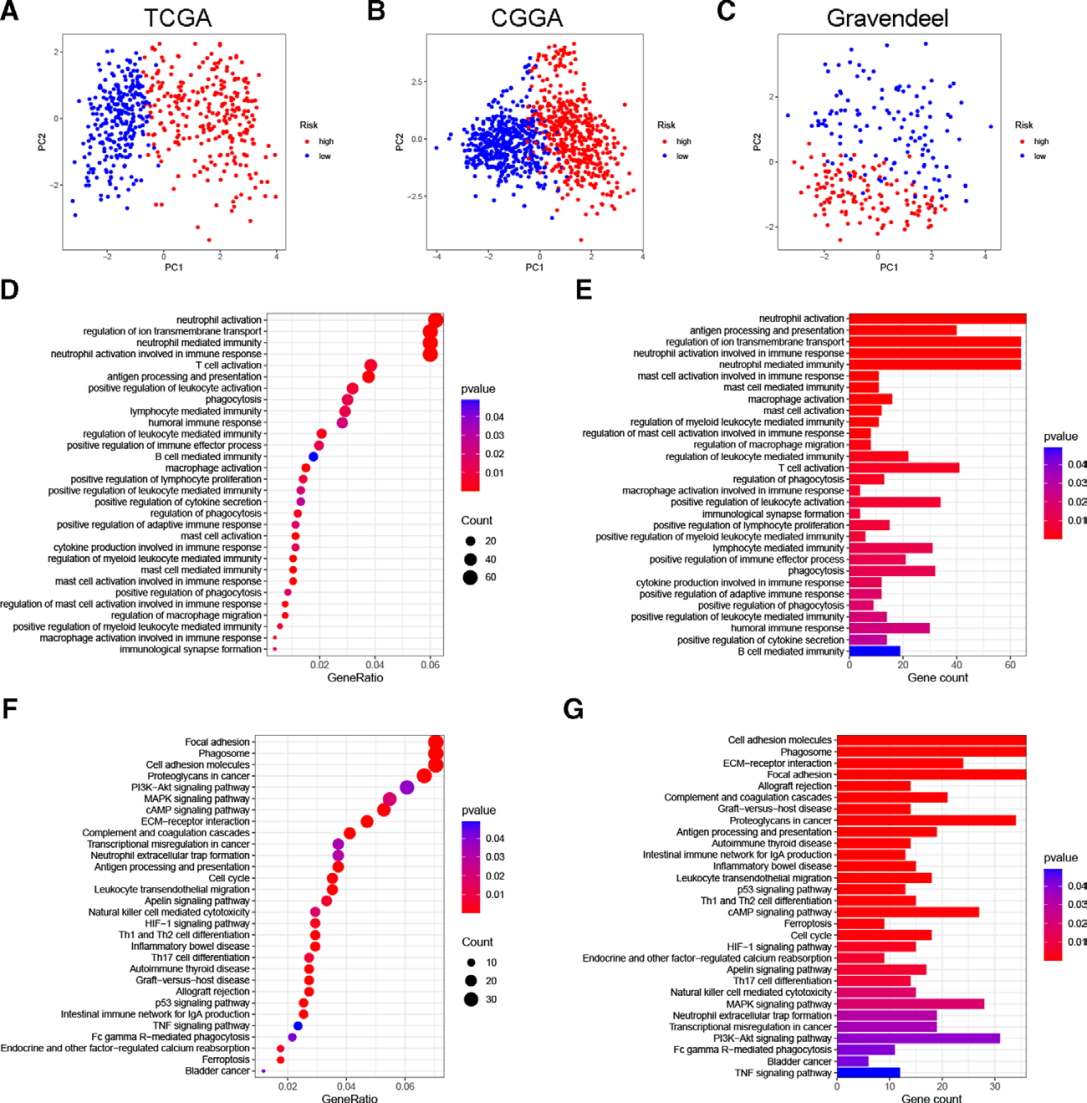
PCA and functional enrichment analysis. (A–C) PCAs were performed according to the expression of signature PRlncRNAs. Representative results of (D–E) biological processes in GO analysis and (F–G) pathways in KEGG analysis. GO = Gene Ontology, KEGG = Kyoto Encyclopedia of Genes and Genomes, PCA = principal component analysis, PRlncRNAs = pyroptosis-related long non-coding RNAs.

Principal component analysis results showed that glioma cases were able to be divided into 2 distinct subgroups based on hub PRlncRNAs (Fig. 5A–C), which demonstrated the good discrimination of the PRLS. Functional enrichment analysis was then carried out in the TCGA cohort to explore PRLS-related biological functions and pathways. Before doing this, we screened 1141 DEGs in the 2 subgroups. Biological processes in Gene Ontology results demonstrated that DEGs were significantly associated with some immune functions (Fig. 5D and E). In Kyoto Encyclopedia of Genes and Genomes results, these DEGs were significantly enriched in cancer-related pathways, including proteoglycans in cancer, PI3K–Akt signaling pathway, MAPK signaling pathway, cell cycle, and ferroptosis (Fig. 5F and G). These results, to some extent, revealed the underlying mechanism of OS differences between the 2 subgroups.

### 3.5. Relationship between the PRLS and immune status

**Figure 6.**
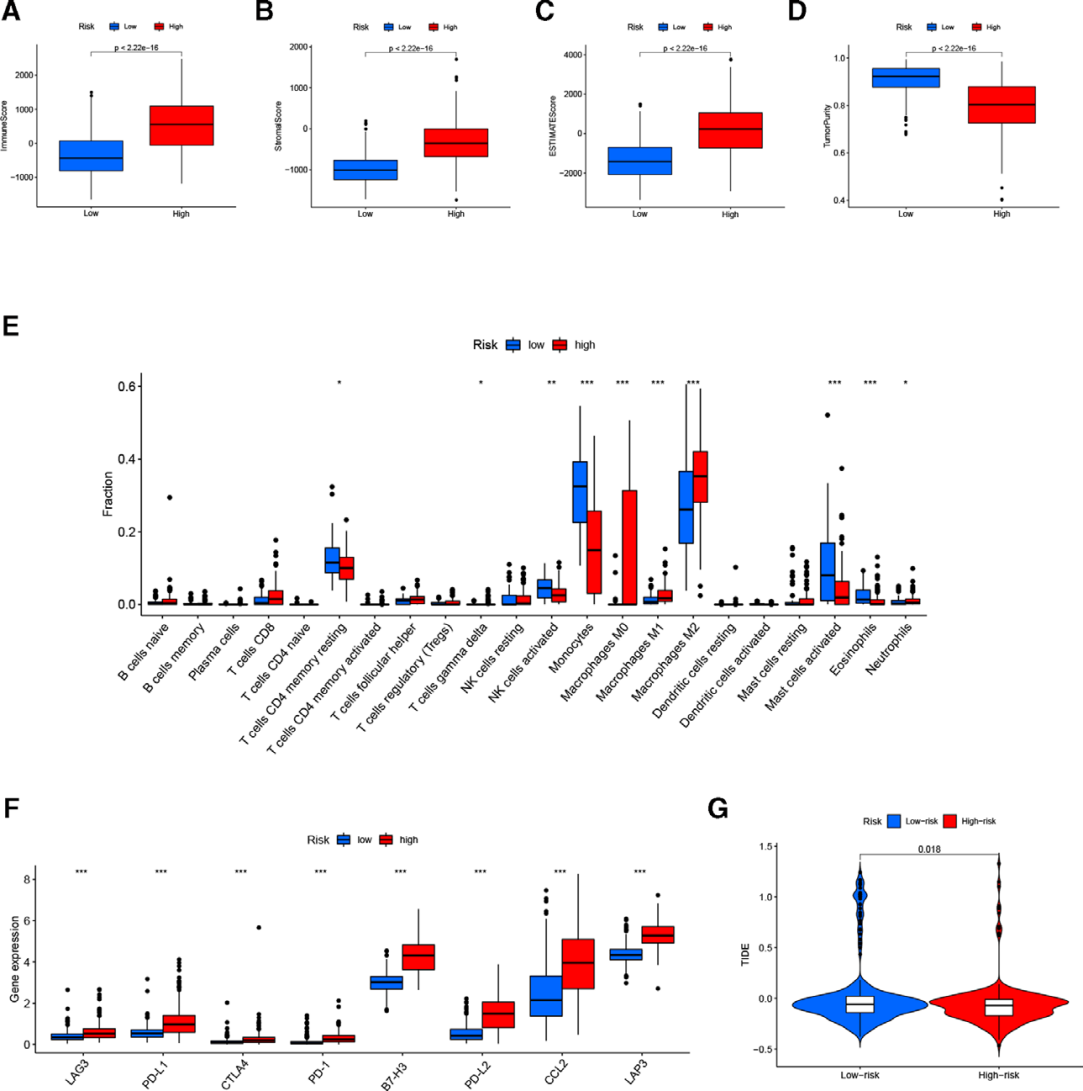
Relationship between the PRLS and immune status in TCGA cohort. (A–D) Correlation of PRLS with immune score, stromal score, ESTIMATE score, and tumor purity. (E) The levels of immune cell infiltration and (F) the expression of immune checkpoints were compared between the 2 glioma subtypes. (G) TIDE prediction scores for 2 glioma subtypes. ***P < .001, **P < .01, *P < .05. PRLS = pyroptosis-related long non-coding RNAs signature, TCGA = The Cancer Genome Atlas, TIDE = Tumor Immune Dysfunction and Exclusion.

We then evaluated the relationship between the PRLS and immune characteristics. Results from the ESTIMATE algorithm indicated that high-risk patients had much higher levels of immune score, stromal score, and ESTIMATE score (Fig. 6A–C), whereas the low-risk patients had greater tumor purity (Fig. 6D). Besides, obvious differences were found in the infiltration levels of 10 immune cells in the 2 subgroups (Fig. 6E). Next, several immune checkpoints was compared in the 2 glioma subtypes. The result suggested that high-risk cases had greater LAG3, PD-L1, CTLA4, PD-1, B7-H3, PD-L2, CCL2, and LAP3 expression levels compared with low-risk cases (Fig. 6F). Finally, using TIDE algorithm, we observed lower TIDE scores in high-risk cases, which means they may be more sensitive to immunotherapy (Fig. 6G).

### 3.6. Association of the PRLS with the TMB and somatic variants

**Figure 7.**
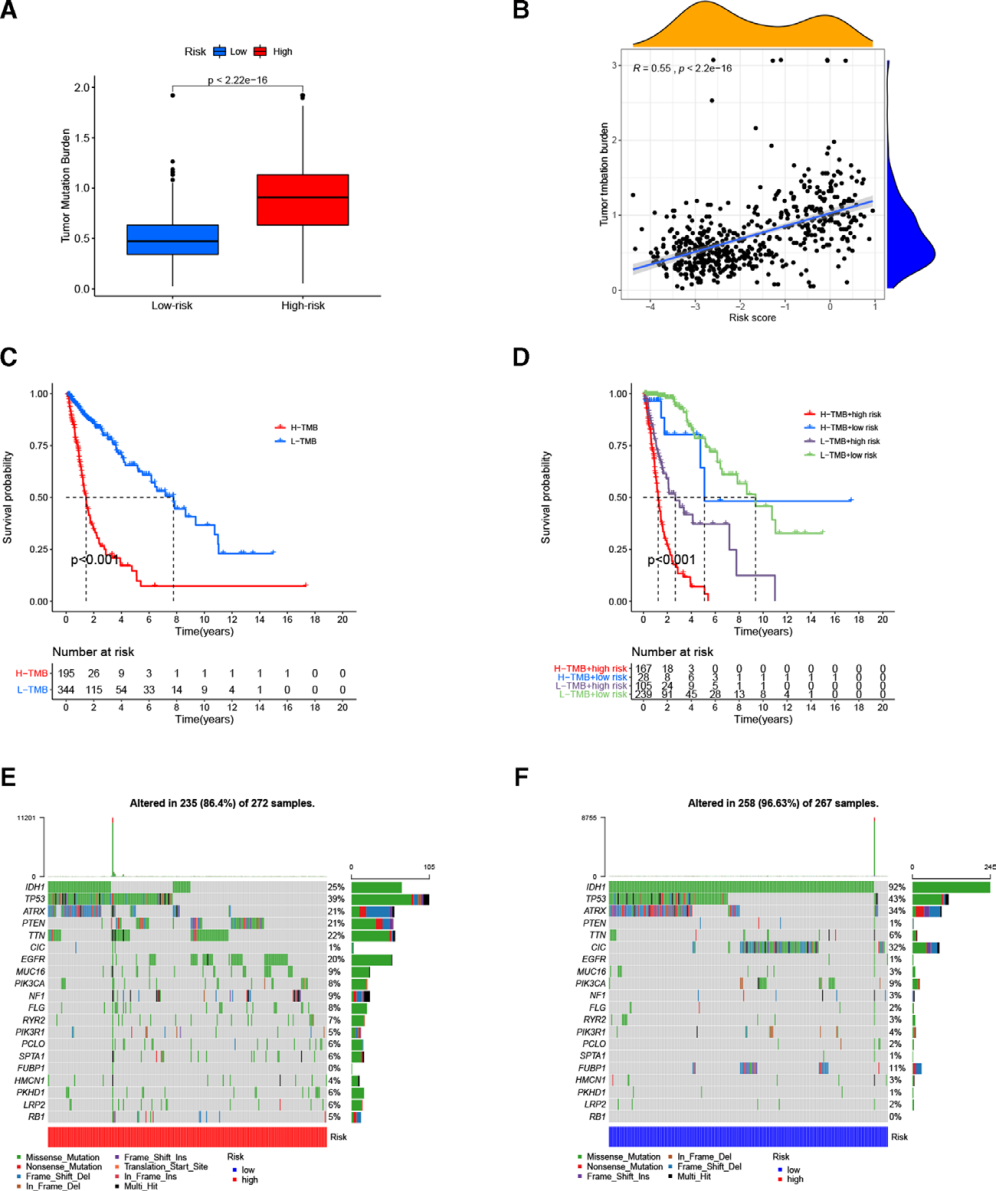
Association of the PRLS with TMB. (A) TMB was compared in the 2 glioma subgroups. (B) Risk score had positive correlation with TMB. (C) The K-M curve for high and low TMB groups. (D) Subgroup survival analysis of PRLS combined with TMB. (E) Waterfall plot of mutation profile in high-risk glioma subgroup. (F) Waterfall plot of mutation profile in low-risk glioma subgroup. K-M = Kaplan–Meier, PRLS = pyroptosis-related long non-coding RNAs signature, TMB = tumor mutation burden.

In the TCGA cohort, high-risk cases had higher TMB levels than low-risk cases (Fig. 7A). Correlation analysis indicated that PRLS score was positively correlated with TMB (*R* = 0.55, Fig. 7B). We then divided the TCGA glioma cohort into the high and low TMB subgroups according to the optimal cutoff point, and found obvious differences in OS of these 2 subgroups (Fig. 7C). Further subgroup analysis revealed that high-risk glioma cases had a shorter OS in the high TMB group, as well as in the low TMB group (Fig. 7D). Additionally, we visualized the landscape of somatic variants in the 2 risk groups through waterfall charts. Waterfall plots show the top 20 genes with the highest mutation frequencies in the TCGA cohort (Fig. 7E and F). Of note, there is recent evidence that TMB can serve as a marker to predict the efficacy of cancer immunotherapy.^[32]^ Thus, our findings further demonstrated that the PRLS might be useful in screening immunotherapy beneficiaries and was reliable in predicting glioma outcomes.

### 3.7. Establishment of a PRLS-based nomogram

**Figure 8.**
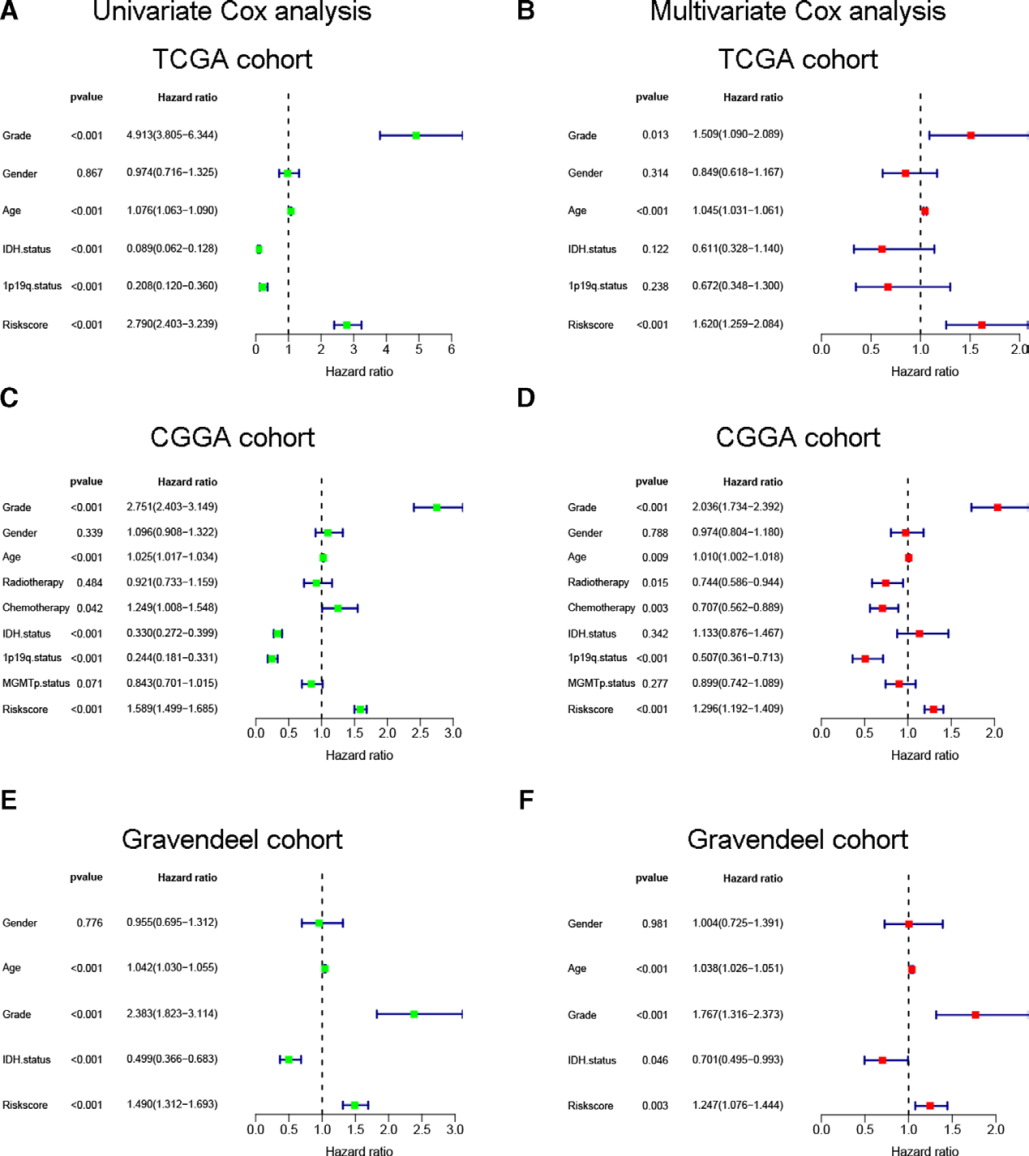
The PRLS was independent of other clinical parameters in predicting glioma patients’ OS. Univariate/multivariate Cox regression analysis was carried out in (A–B) TCGA, (C–D) CGGA and (E–F) Gravendeel cohorts. CGGA = Chinese Glioma Genome Atlas, OS = overall survival, PRLS = pyroptosis-related long non-coding RNAs signature, The Cancer Genome Atlas.

**Figure 9.**
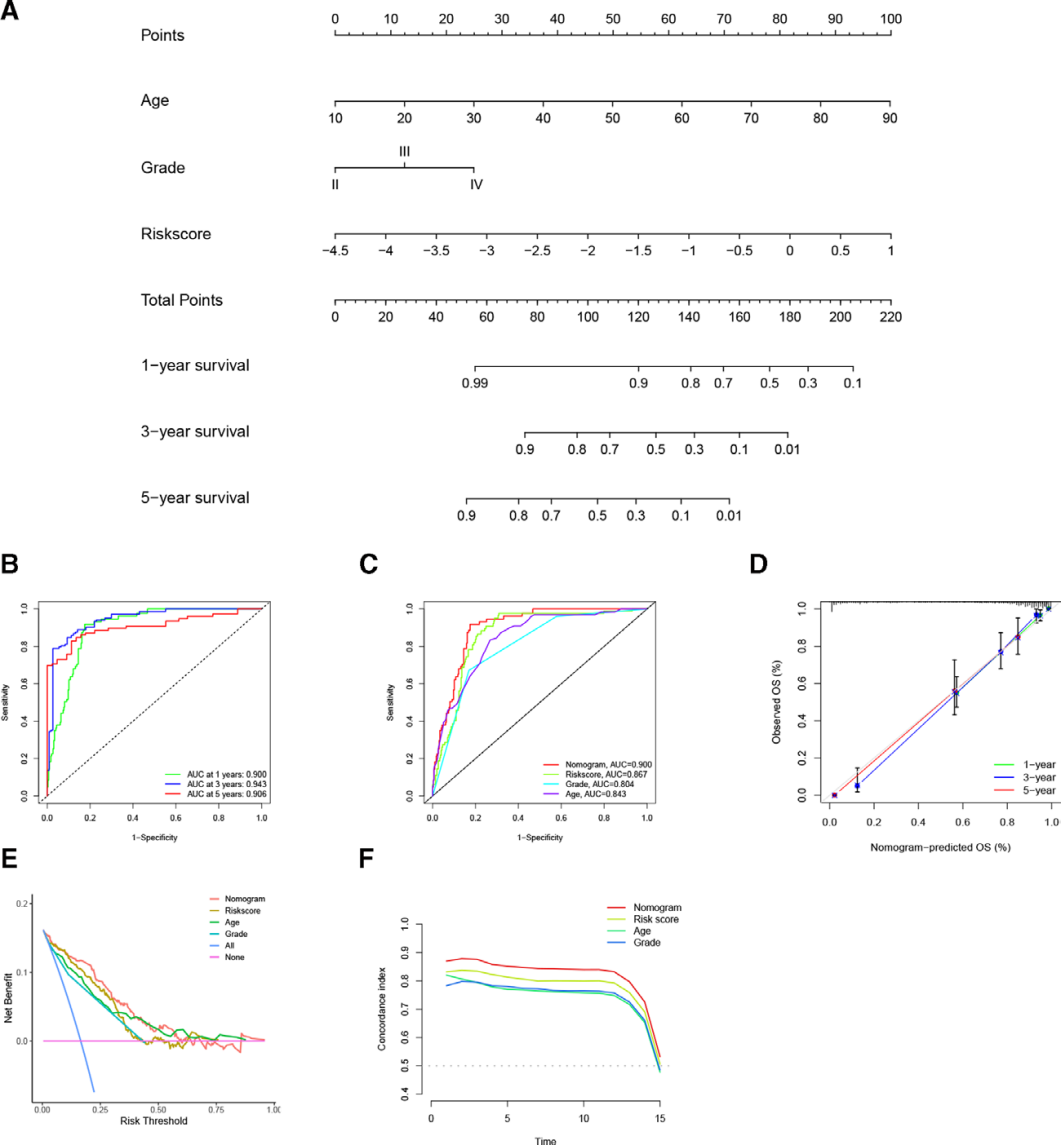
The PRLS-based nomogram building and evaluation. (A) A nomogram containing independent prognostic parameters of TCGA was built. (B) ROC curves for predicting 1 -, 3 -, and 5-year OS based on nomogram scores. (C) ROC curves for the nomogram and independent prognostic parameters. (D) Calibration curves showed that the nomogram’s prediction was close to the actual observation. (E) Decision curve analysis was used to assess the benefit of each parameter in predicting prognosis. (F) The nomogram’s C-index was consistently greater than other independent prognostic parameters. C-index = concordance index, OS = overall survival, PRLS = pyroptosis-related long non-coding RNAs signature, ROC = receiver operating characteristic, TCGA = The Cancer Genome Atlas.

We further evaluated if the PRLS was independent of other clinical parameters in predicting glioma outcomes by univariate/multivariate Cox analysis. Results revealed that the PRLS was an independent risk indicator of glioma in all cohorts (Fig. 8). Next, we bulit a predictive nomogram based on the results of the multivariate analysis (Fig. 9A), and measured its efficacy in a variety of ways. For predicting glioma OS rates, the nomogram had very high area under the curve values (≥0.9), superior to a single prognostic indicator (Fig. 9B and C). Calibration curves showed that the nomogram’s prediction was in good agreement with the actual observation (Fig. 9D). In decision curve analysis, we found that the nomogram provided the greatest benefit in predicting patients’ outcomes (Fig. 9E). Furthermore, the nomogram’s concordance index was consistently greater than other independent prognostic parameters (Fig. 9F). These favorable results highlighted the potential clinical application value of the PRLS-based nomogram.

## 4. Discussion

The existing treatment options for glioma mainly include maximum safe resection, chemotherapy, and radiotherapy, but the curative effect is not satisfactory. Hence, it is urgent to explore more accurate and effective therapeutic markers for glioma. It is noteworthy that biomarkers related to pyroptosis are promising as new therapeutic targets for cancer.^[33]^ Recently, a variety of cancers have been reported to be sensitive to pyroptosis, such as colon, liver and breast cancers.^[34–36]^ Numerous lncRNAs have also been confirmed to participate in pyroptosis and regulate the biological behavior of tumors.^[37–39]^ As a result, many researchers attempted to use transcriptome data to systematically evaluate the relationship of PRlncRNAs with cancer prognosis, immunity, and therapeutic response. Recent studies suggest that the PRLS is satisfactory in predicting the prognosis of several cancers.^[21,23,24,40]^ These studies provide new clues for further research on the role of PRlncRNAs in cancer.

In this study, we selected share prognostic PRlncRNAs from 3 glioma cohorts to ensure lncRNAs used to build the PRLS were universal. Based on the TCGA cohort, we further identified hub PRlncRNAs by LASSO and multivariate Cox regression analyses, and developed a 7-PRlncRNA prognostic signature. K-M and tdROC curves demonstrated that our PRLS predicted glioma patients’ outcomes with high accuracy in all cohorts. Moreover, our data suggested that the PRLS had a stable predictive power even in patients who had received radiotherapy or chemotherapy. In clinical correlation analysis, glioma subtypes with poor outcomes (including advanced age, higher grade, IDH wild-type, and 1p19q non-codeletion) were mainly concentrated in the high-risk group. Moreover, univariate/multivariate Cox analysis further demonstrated that our PRLS could predict patients’ OS independently of other clinical indicators. Next, using independent prognostic parameters, a nomogram was built and showed greater efficiency in predicting survival.

Our PRLS was composed of 7 lncRNAs, including CRNDE, FAM66C, HAR1A, HOTAIRM1, LINC00092, LINC00173, and LINC00641. Previous studies have shown that most of them are closely linked to the tumorigenesis and progression of gliomas. For example, CRNDE is upregulated in gliomas, and CRNDE overexpression predicts high malignant level and poor clinical prognosis of glioma.^[41]^ FAM66C is downregulated in glioma tissue, and its deletion can significantly promote cell proliferation and migration, which is associated with the regulation of miRNA/LATS1 signaling pathway.^[42]^ HOTAIRM1, as an oncogenic lncRNA in gliomas, is linked to a variety of malignant progression of gliomas.^[43,44]^ Based on function enrichment analyses, DEGs were observed to be closely linked to various immune-related functions and pathways. Furthermore, the immune microenvironment and levels of immune cell infiltration exhibited obvious differences in the 2 subgroups. In recent years, the immunotherapy represented by ICIs has brought a new revolution to the treatment of cancer patients. However, how to screen the best beneficiaries has always been the focus of immunotherapy research, and the development of biomarkers is one of the major research directions.^[45,46]^ Currently, the ICIs therapy can effectively improve the prognosis of various cancers, and the immune checkpoint expression has become an important indicator of ICIs therapy.^[47]^ TIDE algorithm is a novel method to predict treatment response to ICIs, which can replace a single biomarker. Patients with low TIDE scores are generally considered to have a better immunotherapy response.^[30]^ TMB is another well-studied immunotherapy biomarker. Compared with PD-L1 expression, which does not predict ICIs responses such as melanoma, TMB is a more easily assessable predictive biomarker that could be incorporated in the researches of all solid malignant tumor.^[48]^ The early progress has been made in the use of TMB to predict the responses to ICIs in cancer patients.^[49,50]^ Evidence suggests that high TMB is an independent biomarker of ICIs response in multiple tumor types. The likelihood of ICI benefits increases as TMB increases.^[51]^ In our study, high-risk cases had greater levels of immune checkpoints and TMB, as well as lower TIDE scores, suggesting that high-risk patients were potential beneficiaries of immunotherapy. Hence, our PRLS may be an effective marker for predicting the immunotherapy response of glioma.

Undoubtedly, the limitations in our study should be considered. The data we analyzed were from public databases, and the prognostic role of 7 hub PRlncRNAs in glioma and their role during pyroptosis need to be further verified by clinical and basic experimental data. In addition, prospective trials used to assess the predictive power of our PRLS for immunotherapy response will be more convincing.

## 5. Conclusions

In summary, we built a 7-PRlncRNA signature for glioma, which proved to be a good predictor of clinical outcome and immunotherapy response. Our findings may provide insights into clinically individualized treatment strategies for glioma.

## Author contributions

**Conceptualization:** Ligen Mo, Teng Deng.

**Data curation:** Fangzhou Guo.

**Formal analysis:** Teng Deng.

**Methodology:** Qianrong Huang, Teng Deng.

**Software:** Qianrong Huang, Jun Yan.

**Visualization:** Jun Yan, Qian Jiang.

**Writing – original draft:** Qianrong Huang, Jun Yan.

**Writing – review & editing:** Teng Deng.

## Supplementary Material


