# Supplementary material for: Construction of a pyroptosis-related lncRNAs signature for predicting prognosis and immunotherapy response in glioma
Source: Medicine (Baltimore). 2023 Feb 10;102(6):e32793. doi: 10.1097/MD.0000000000032793 (PMC9907962; doi:10.1097/MD.0000000000032793)
Supplement: Supplementary file 1 [file medi-102-e32793-s001.pdf]

Supplementary Table 1. Clinical and molecular characteristics of glioma cases in the 3 cohorts

Characteristics		TCGA (n = 553)	CGGA (n = 961)	Gravendeel (n = 251)
Age				
	< 50	305	673	121
	≥ 50	248	287	130
	NA	0	1	0
Gender				
	Male	319	564	167
	Female	234	397	84
WHO grade				
	II	196	269	23
	III	212	321	81
	IV	145	371	147
IDH status				
	Mutant	340	496	76
	Wild-type	207	416	125
	NA	6	49	50
1p19q codeletion status				
	Codeletion	138	199	NA
	Non-codeletion	410	691	NA
	NA	5	71	NA
MGMTp status				
	Methylated	NA	453	NA
	Un-methylated	NA	355	NA
	NA	NA	153	NA
Radiotherapy				
	Yes	NA	737	NA
	No	NA	190	NA
	NA	NA	34	NA
Chemotherapy				
	Yes	NA	666	NA
	No	NA	262	NA
	NA	NA	33	NA
